# Thermal Degradation Mechanism and Decomposition Kinetic Studies of Poly(Lactic Acid) and Its Copolymers with Poly(Hexylene Succinate)

**DOI:** 10.3390/polym13091365

**Published:** 2021-04-22

**Authors:** Iouliana Chrysafi, Nina Maria Ainali, Dimitrios N. Bikiaris

**Affiliations:** 1 Laboratory of Advanced Materials and Devices, Department of Physics, Faculty of Sciences, Aristotle University of Thessaloniki, GR-54124 Thessaloniki, Greece; iochrysa@physics.auth.gr; 2 Laboratory of Polymers Chemistry and Technology, Department of Chemistry, Faculty of Sciences, Aristotle University of Thessaloniki, GR-54124 Thessaloniki, Greece; ainali.nina@gmail.com

**Keywords:** poly(lactic acid), poly(hexylene succinate), block copolymers, thermal degradation kinetics, thermal stability, pyrolysis, mechanical properties

## Abstract

Ιn this work, new block poly(lactic acid)-block-poly(hexylene succinate) (PLA-b-PHSu) copolymers, in different mass ratios of 95/05, 90/10 and 80/20 *w*/*w*, are synthesized and their thermal and mechanical behavior are studied. Thermal degradation and thermal stability of the samples were examined by Thermogravimetric Analysis (TGA), while thermal degradation kinetics was applied to better understand this process. The Friedman isoconversional method and the “model fitting method” revealed accurate results for the activation energy and the reaction mechanisms (nth order and autocatalysis). Pyrolysis-Gas Chromatography/Mass Spectrometry (Py-GC/MS) was used to provide more details of the degradation process with PHSu’s mechanism being the *β*-hydrogen bond scission, while on PLA the intramolecular *trans*-esterification processes domains. PLA-b-PHSu copolymers decompose also through the *β*-hydrogen bond scission. The mechanical properties have also been tested to understand how PHSu affects PLA’s structure and to give more information about this new material. The tensile measurements gave remarkable results as the elongation at break increases as the content of PHSu increases as well. The study of the thermal and mechanical properties is crucial, to examine if the new material fulfills the requirements for further investigation for medical or other possible uses that might come up.

## 1. Introduction

Poly(lactic acid) (PLA) is one of the most popular polymers, gaining more and more attraction from research, medical and industrial fields, due to its biocompatibility, degradability, non-toxicity and easy processing [1]. It is an aliphatic polyester, produced by industrial polycondensation of lactic acid and/or ring-opening polymerization of lactide. Although in literature, PLA is considered as a biodegradable polymer [2], its biodegradability appears under specific industrial composting conditions [3] or by introducing another bio-based material in its matrix [4,5].

The biocompatible nature of PLA makes it suitable for numerous biomedical applications such as in wound management and stents, in orthopedic and fixation devices, in drug delivery systems and in tissue engineering and regenerative medicine [6]. For each application different physical, mechanical, chemical and degradation properties are required. Το select a PLA-based polymer for medical applications, the general criterion is to match mainly the mechanical properties and the degradation time to the needs of the specific application. For example, PLA struts should be thicker than metal to obtain the desired radial strength, but that may lead to poor deliverability, platelet de-position or vessel injury. Thus, the mechanical response of PLA implant/device should thoroughly be examined [6]. The high strength of PLA and the ability to transfer stress overtime to the damaged area, allowing the healing of the tissues make it acceptable in orthopedics. For scaffolds, the main criterion is to maintain their mechanical properties as long as they are needed before they are finally excreted by the body [7]. PLA has also been extensively investigated for drug delivery, as due to its nature it undergoes hydrolysis into the human body. The biodegradation products (lactic acid, glycolic acid) do not affect the normal cell function and eventually are removed from the body [8]. Neat PLA and various blends and copolymers with PLA in the form of micro, nano-particles, polymeric micelles or polymersomes have been used to encapsulate different drugs such as hormones, psychotic, restenosis, oridonin and dermatotherapy [6,9].

PLA is a very brittle material; it exhibits high tensile strength and low elongation at break (about 5%) and consequently has high modulus and rapid degradation rate making it more suitable for drug delivery systems than for other applications [6]. Additionally, it is susceptible to thermal degradation during processing. In fact, the thermal degradation of PLA is a complex phenomenon and may be credited to different mechanisms. Moreover, other significant variables can impact the thermal degradation of PLA like monomers, oligomers, crystallization, molecular weight distribution and metal catalysts [10,11,12,13,14]. A detailed kinetic analysis of the thermal degradation of PLA has been many times presented in literature in comparison with the respective blends or copolymers of PLA and its activation Energy (E_a_) was found. So far and as we know only Chrissafis [15] studied the reaction models through kinetic analysis resulting three kinetic models (Cn, Bna and Fn) with quite equal results. Monika [16] applied the Ozawa-Flynn-Wall (OFW) (E_a_ = 131 kJ/mol) and Kissinger (E_a_ = 132.9 kJ/mol) isoconversional methods. Yuzay [17] used also the same methods as Monika and unlike Monika observed constant activation energy around 115 kJ/mol approximately. Das [18] applied 5 different isoconversional methods obtaining a quite constant activation energy compared to the degree of conversion with the E_a_ values fluctuating between 97 and 130 kJ/mol. He also applied the AIC method with not as accurate results as the model fitting method. Shanshan [10] calculated the E_a_ through the OFW method and its value starts at 146 kJ/mol for a = 0.1 and arrives at 192 kJ/mol for a = 0.9. Concluding, due to literature, the activation energy of PLA is mostly calculated by the OFW method and its value fluctuated between 110 and 195 kJ/mol, while in comparison with the degree of conversion it can be either constant or increasing as the degree of conversion as well. Τo modify the thermal and mechanical properties to be suitable for each application separately, many additives such as other polymers or inorganic fillers have been used to form new blends or copolymers [19]. Poly(lactic acid-co-glycolic acid) (PLGA), is one of the most used copolymers in drug delivery [20,21] which is approved by the Food and Drug Administration (FDA). l-lactide copolymers with polycaprolactone (PCL) aim to alter the l-lactide crystallinity accelerating the degradation process or include plasticizers to increase the ductility [6]. PLA-poly(hydroxy butyrate) (PHB) and PLA-poly(ethylene glycol) (PEG) blends improve PLA’s mechanical properties [22,23]. Research in this field is widespread and new scientific results are appearing daily to find new systems that will improve the mechanical properties of PLA and more.

Succinic acid is widely used in chemistry, pharmacy and in food industries. It can be produced by fermentation but for industrial use, its bio-based forms come into conflict with its petrochemical synthesis due to its cheaper manufacturing cost [24]. Nevertheless, the fermentative production of succinic acid is gaining ground due to the ascending concerns of the global greenhouse effect and the limited fossil resources [25]. Succinic acid is non-toxic, soluble in water and can be easily converted to other bulk chemicals such as 1,4-butanediol, gamma-butyrolactone, tetrahydrofuran, adipic acid, n-methylpyrrolidone or linear aliphatic esters [26,27]. Except for their excellent physicochemical properties, these copolyesters provide biodegradability, biocompatibility and easy compostability, predisposing that their good properties will follow to other succinic acid forms [28,29].

Poly(hexylane succinate) PHSu was selected as a promising material, to introduce a new optimized copolymer for medical applications, which is not thoroughly studied as such in literature. According to Bai Z. [30] PHSu presents the highest degradation rate compared to poly(ethylene succinate) (PES) and poly(butylene succinate) (PBS). Moreover, so far, only Lostocco and Huang [31,32] have investigated the effect of PHSu on poly(lactic acid) structure in the form of blends through gel-permeation chromatography for molecular weight studies and differential scanning calorimetry for degradation studies after hydrolysis. In addition, as far as we know, the study of the thermal degradation kinetics, the calculation of the activation energy E, the pre-exponential factor A and the reaction model f(a) is for the first time introduced in literature. Thus, a full investigation of the new copolymers’ physicochemical properties and the study of their behavior in different environments is crucial.

This work aims to study the thermal and mechanical properties as well as the kinetic mechanism of new PLA-b-PHSu copolymers compared to neat PLA, and investigate the influence on their thermal stability, degradation and mechanical properties. When a new material is synthesized its general properties should be thoroughly examined, not only to verify whether it has all the requirements for the specific application and further investigation but also to explore new possible uses for different applications. Thermogravimetric analysis (TGA) was used in conjunction with pyrolysis-gas chromatography/mass spectroscopy (Py-GC/MS) to provide more details about the degradation process. Py-GC/MS provides detailed information about the thermal degradation pathways of polymeric materials. A thorough investigation of the degradation mechanism and the decomposition products can allow the selection of optimized strategies required to control thermal stability. Knowing the above parameters, it is possible to understand the nature of the processes and the effect of the copolymers on the degradation products. Thus, it would be easier to modify the copolymers to improve their properties. The results from the kinetic analysis and Py-GC/MS are reported for first time in literature. In addition, the tensile mechanical properties were studied in order to investigate possible improvements in tensile stress and elongation at break with increasing the content of PHSu. Last but not least, thermal degradation kinetics was applied to delve into the degradation mechanism and analyze the thermal degradation process, while useful information about the activation energy E, pre-exponential factor A and the reaction model f(a) were obtained.

## 2. Materials and Methods

### 2.1. Materials

l-lactide (98+%) (S,S)-3,6-Dimethyl-1,4-dioxane-2,5-dione, was obtained from Alfa Aesar Chemicals (Kandel, Germany). Succinic acid (ACS reagent, ≥99.0%), 1,6-hexanediol (99%), tetrabutyl titanate (TBT) (97%) and Tin(II) 2-ethylhexanoate (TEH) (96%) catalysts, were purchased from Sigma-Aldrich chemical company (Saint Louis, MO, USA). The other solvents or reagents that have been used were of analytical grade and obtained also from Sigma-Aldrich.

### 2.2. Synthesis of (PLA-b-PHSu) Copolymers

PHSu was synthesized by the two-stage melt polycondensation method, including first the esterification, where PHSu oligomers were synthesized and then the second stage of polycondensation, where the final product was obtained. PLA-b-PHSu copolymers in 95/5, 90/10 and 80/20 mass ratios, were synthesized by ring opening polymerization (ROP) of L-lactide [33]. TBT catalyst was used in the synthesis of PHSu, while TEH in the synthesis of the copolymers. As was in a previous work measured by size exclusion chromatography (SEC), the prepared copolymers have number average molecular weight values (*M_n_*) 44,000, 33,000 and 21,700, respectively, while neat PLA has 47,000and PHSu 5000 [33].

### 2.3. Characterization Methods

#### 2.3.1. Tensile Mechanical Properties

An Instron 3344 dynamometer was used to perform the measurements of the tensile mechanical properties of the copolymers and neat PLA, in accordance with ASTM D638 (Norwood, MA, USA), using a crosshead speed of 10 mm/min and dump-bell-shaped molds (central portions 5 mm × 1 mm thick, 22 mm gauge length). In order to prepare the samples for the Instron, each sample (PLA, PLA-b-PHSu95/05, PLA-b-PHSu90/10 and PLA-b-PHSu80/20) placed in a PW 30 Otto Weber (Dusseldorf, Germany) hydraulic press connected to a temperature controller (Omron E5AX, Dusseldorf, Germany). The samples were heated above their melting temperature, then left in room temperature to cool down and lastly removed from the molds. Five specimens of each copolymer and neat PLA were tested to ensure reproducibility by obtaining mean values. From the stress-strain curves, the values of tensile strength at the yield point and break, elongation at break and the Young modulus were calculated. To have a general view of the samples, their degree of crystallinity (Xc) as obtained from Differential Scanning Calorimetry (DSC) experiments is also quoted. PLA presents the highest value with Xc = 36%, then PLA-b-PHSu95/05 with Xc = 32%, PLA-b-PHSu90/10 with Xc = 28% and PLA-b-PHSu80/20 with Xc = 27% (heating rate 10 °C/min) [33].

#### 2.3.2. Thermogravimetric Analysis (TGA)

Thermogravimetric analysis of PLA, PHSu and their 3 copolymers was performed using a SETARAM SETSYS TG-DTA 16/18 instrument (Setaram Instrumentation, Lyon, France). The samples (5 ± 0.5 mg) were placed in alumina crucibles and heated from RT to 600 °C, in a 50 mL min^−1^ flow of N_2_ at heating rates of 5, 10, 15 and 20 °C/min, an empty alumina crucible was used as reference. Continuous recordings of sample temperature, sample mass, its first derivative and heat flow were taken. Kinetic analysis of the thermal decomposition process, in order to calculate the values of the kinetic triplet (activation energy E_a_, pre-exponential factor A and reaction model f(a)) was performed by the NETZSCH Kinetics Neo software (Netzsch, Wunsiedel, Germany).

#### 2.3.3. Pyrolysis-Gas Chromatography/Mass Spectrometry (Py-GC/MS) Study

For Py-GC/MS analysis of the copolymers, a very small amount of each material was “dropped” initially into the ‘Double-Shot’ EGA/PY 3030D Pyrolyzer (Frontier Laboratories Ltd, Fukushima, Japan) using a CGS-1050Ex carrier gas selector. For pyrolysis analysis (flash pyrolysis), each sample was placed into the sample cup which afterwards fell free into the Pyrolyzer furnace. The pre-selected pyrolysis temperature was differentiated for each sample, while the GC oven temperature was programmed at 50 °C for 2 min, followed by a stepped increase to 200 °C with a heating rate of 5 °C/min, where it was held for 8 min, and then the temperature was increased at 300 °C by a rate 20 °C/min, where it was held for 5 min. The mass selective detector was programmed to detect masses between 35 and 500 amu (atomic mass unit). Sample vapors generated in the furnace were split (at a ratio of 1/50), a portion moved to the column at a flow rate of 1 mL/min, pressure 53.6 kPa and the remaining portion exited the system via the vent. The pyrolyzates were separated using temperature programmed capillary column of a Shimadzu QP-2010 Ultra Plus (Kyoto, Japan) gas chromatogram and analyzed by the mass spectrometer MS-QP2010SE of Shimadzu (Kyoto, Japan). Ultra-ALLOY® metal capillary column from Frontier Laboratories LTD (Fukushima, Japan) was used containing 5% diphenyl and 95% dimethylpolysiloxane stationary phase, column length 30 m and column ID 0.25 mm. For the mass spectrometer, the following conditions were used: Ion source heater 200 °C, interface temperature 300 °C, vacuum 10^−4^ – 10^0^ Pa, m/z range 35–500 amu and scan speed 10,000. The ion gas chromatograms and spectra retrieved by each experiment were subjected to further interpretation through Shimadzu and Frontier post-run software. The chromatogram and spectra retrieved by each experiment were subject to further interpretation through Shimadzu (NIST11.0) and Frontier (F-Search software 4.3) post-run software. Identification was recognized depending on the similarity percentage (minimum value of 80%) between average mass spectra on the entire chromatogram. Moreover, Kovats retention indices [34] were also calculated for the thermal degradation products in order to compare these values with the data of the NIST library and fulfil a reliable identification of the pyrolyzates (not shown for briefness).

## 3. Results and Discussion

### 3.1. Tensile Mechanical Properties

**Figure 1:**
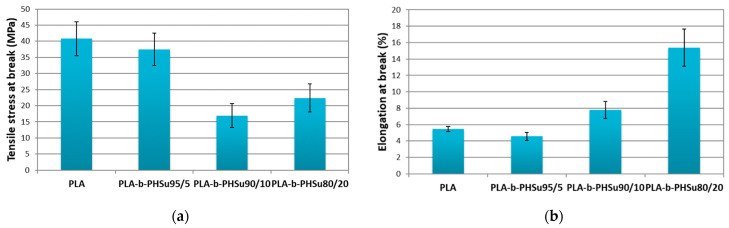
Comparison for (**a**) tensile stress at break and (**b**) elongation at break for neat PLA and PLA-b-PHSu copolymers. (mean ± st.dev, *n* = 8).

The detailed synthesis of PLA-b-PHSu copolymers described in our previous work and their chemical structure was proved by Fourier-transform infrared spectroscopy (FTIR) and Nuclear magnetic resonance (NMR) [33]. The mechanical properties of neat PLA and its PHSu copolymers of various concentrations were studied by tensile tests. Figure 1a shows the tensile strength at break and Figure 1b the elongation at break values of the copolymers compared to neat PLA. The bars present the standard deviation. Neat PLA showed characteristics of glassy polymers with low deformation at break, while its tensile stress value is the largest and the elongation at break is almost the smallest. Similar values for PLA were reported by Navarro-Baena, Abdelwahab and Mittal [23,35,36]. As expected, the tensile stress at break of the copolymers is smaller compared to neat PLA due to the brittle structure of PHSu, which was attributed to its low molecular weight (5000 g/mol) [31]. The values of PLA-b-PHSu95/05 are close to those of PLA, due to the small amount of PHSu. The values of PLA-b-PHSu90/10 fluctuate between those of the other two copolymers. As for PLA-b-PHSu80/20, it exhibits the highest value of elongation at break compared to the other copolymers and in fact it is three times higher than PLA (Figure 1b). Kumar Mohapatra [37] synthesized PLA/PEG blends and noticed the same pattern; as the content of PEG increased the tensile stress was getting lower and the elongation at break higher.

### 3.2. Thermogravimetric Analysis (TGA)

**Figure 2:**
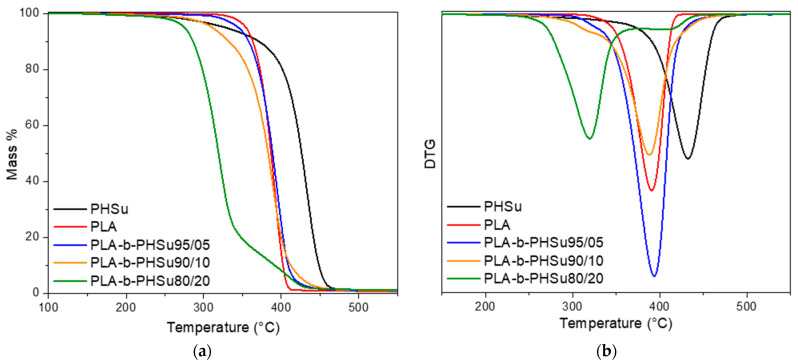
(**a**) Mass (%) and (**b**) DTG versus temperature curves obtained from TGA experiments Figure 3. copolymers at the heating rate of 20 °C/min.

Thermal degradation of PHSu, PLA and their copolymers was studied by TGA experiments. Figure 2 shows the mass (%) and the derivative of the mass loss (DTG) versus temperature at the heating rate of 20 °C/min under nitrogen atmosphere. As it can be seen from Figure 2, the curves of PHSu, PLA and the 3 copolymers do not present the same pattern, with the curve of PHSu starting first to lose mass at around 180–200 °C, but the main mass loss step is shifted to higher temperatures compared to the other samples. PLA presents one mass loss step which can be simultaneously seen in the mass (%) and in the DTG curve and is associated with the loss of ester groups by unzipping depolymerization [38]. As far as copolymers are concerned, PLA-b-PHSu95/05 follows quite the same pattern as pure PLA, as the content of PHSu is quite small. In PLA-b-PHSu90/10, the mass loss procedure starts at lower temperature (around 190–200 °C) and two mass loss steps can be seen, even though the first one is too small. PLA-b-PHSu80/20 has the largest differences compared to the other copolymers; it starts to lose mass at approximately 180 °C, around 90 °C sooner than PLA and in much lower temperature than all copolymers. Based on these results, it is assumed that PHSu content affects the mass loss behavior of copolymers and the first decomposition step could be attributed to the sample’s oligomers that derive from the low molecular weight of PHSu.

A better view of the samples’ behavior can be seen in Figure 2b. From the DTG curves, it can be inferred that the degradation of neat polymers is carried out as a one-step process, as only one peak is observed at approximately 390 °C for PLA and at 432.3 °C for PHSu. PLA-b-PHSu95/05 and PLA-b-PHSu90/10 present two degradation steps; the first is quite small and is affected by the presence of PHSu on the polymeric matrix and the second is almost in the same temperature with PLA and is the main decomposition peak. A completely different trend can be observed on PLA-b-PHSu80/20 copolymer, where there are also 2 degradation steps, but the first corresponds to the main decomposition step and the second to the minor mass loss. The main degradation step occurs at lower temperatures as it was also observed in the mass loss curve. It cannot be assumed that the first step is due to PHSu, because PHSu is the minor component, only 20%, while the PLA block is the majority. Thus, the second step would be expected to be to a higher extent as in the other two copolymers. However, it can be seen that as the content of PHSu increases in copolymers, the extent of the first degradation step increases too, while the temperature that this mass loss takes place at is shifted to progressively lower values. Table 1 presents the temperatures of the first and the maximum degradation rate peaks of copolymers recorded by TGA.

**Figure 3:**
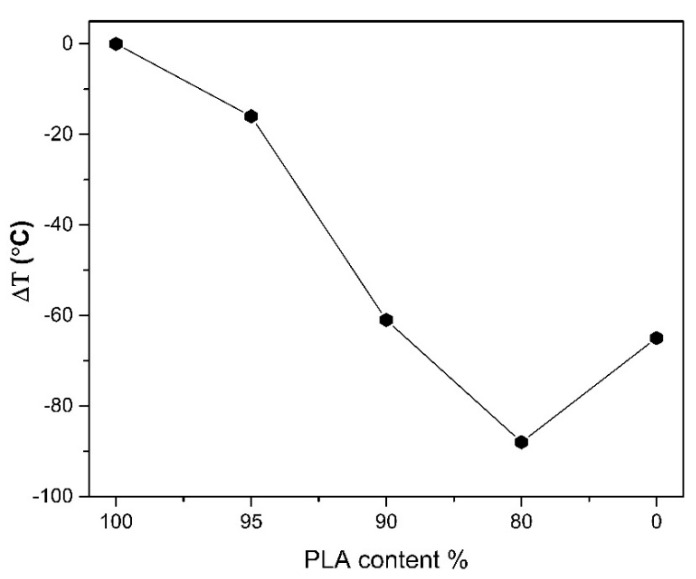
Thermal stability variation of PHSu and PLA-b-PHSu copolymers compared to neat PLA, using 2% mass loss as a comparison point.

Thermal stability of PLA, PHSu, PLA-b-PHSu80/20, PLA-b-PHSu90/10 and PLA-b-PHSu95/5 copolymers based on 2% of mass loss, is given in Figure 3 and is calculated compared to neat PLA. PLA is the most thermally stable material and as the content of the PHSu increases, the copolymers are less stable. Moreover, it can be observed that PLA-b-PHSu80/20 is the least thermally stable material even from PHSu. This phenomenon, as mentioned above, is possibly due to the low molecular weight of the sample or due to the accelerating effect of PHSu. Mamun [39] observed that in poly(ε-caprolactone)/polystyrene (PCL/PS) blends with low PS molecular weight, the sample was less stable than that of high PS molecular weight. In addition, Chrissafis [40] noticed that in the low molecular weight poly(ethylene succicate) the mass loss curve presents two degradation steps, with the first being affected by the early decomposition of the oligomers. Although PHSu is less thermally stable than PLA, it can be inferred that it is the most stable at higher temperatures between 340 °C and 380 °C, where the degradation of PLA and its copolymers is already in “half way”. Therefore, it seems that the addition of high amounts of PHSu, accelerates the decomposition of PLA. For this reason, to further investigate the degradation process, Py-GC/MS was applied to the two degradation peak temperatures of PLA-b-PHSu80/20 and generally to PLA, PHSu and to the two other copolymers with the results being later discussed.

### 3.3. Pyrolysis-Gas Chromatography/Mass Spectrometry Study

**Figure 4:**
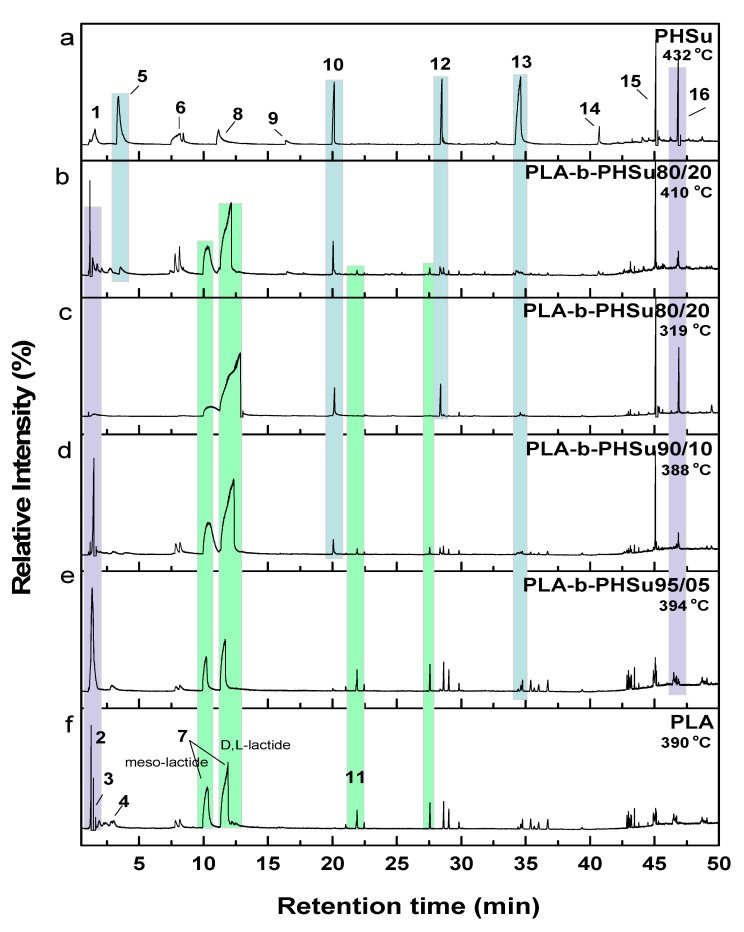
Total ion gas chromatograms of (**a**) neat PHSu, (**b**–**e**) PLA and PHSu copolymers and (**f**) neat PLA (light blue areas highlight the peaks corresponding to PLA segment, while yellow areas demonstrate the PHSu’s segments).

In order to fully explore the degradation process of the studied polymers, temperatures corresponding to each sample’s degradation peak were selected for Py-GC/MS analysis (Table 1). In fact, Py-GC/MS provides detailed information about the thermal degradation pathways of polymeric structures, allowing though the selection of optimized parameters required for thermal stability control. Herein, all copolymers were pyrolyzed at a temperature range of 319–432 °C, while the recorded chromatograms of the degradation products are shown in Figure 4. The most prominent pyrolysis products were identified through their MS spectra (not shown for briefness) after the comparison with mass-spectra libraries and literature data, and are presented in Table 2.

**Scheme 1:**
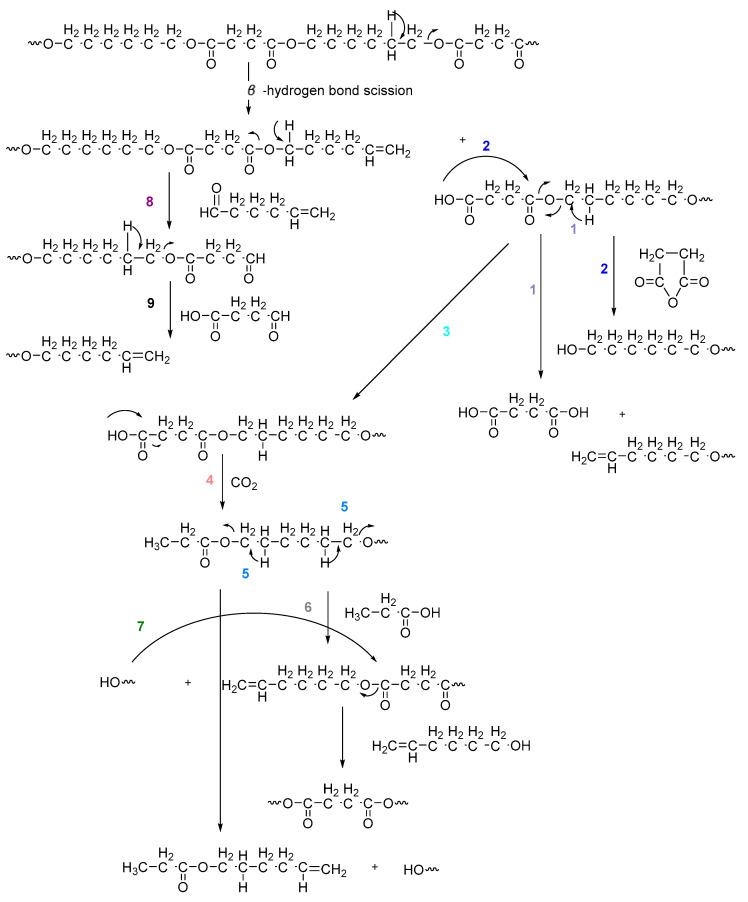
Proposed mechanism of PHSu thermal degradation.

On the one hand, the chromatogram of PHSu (Figure 4a) presents a simple pattern, showing a few major peaks which are identified as acetaldehyde or carbon dioxide, 1,5-hexadiene, succinic acid or succinic anhydride, 1,6-hexanediol, 5-hexen-1-ol, hex-5-en-1-yl-4-hydroxybutanoate, hex-5-en-1-yl propionate, 4-((6-hydroxyhexyl)oxy)-4-oxobutanoic acid, bis(6-hydroxyhexyl) succinate, 4-oxo-4-((6-((4-oxobutanoyl)oxy)hexyl)oxy)butanoic acid. The identified structures could be classified into vinyl-, hydroxyl-, methyl-, carboxyl- and aldehyde-terminated compounds. Scheme 1 presents the proposed mechanism of PHSu degradation. Regarding the principle thermal degradation pathway of carboxylic esters, it is composed of a *β*-hydrogen transfer rearrangement, leading to the formation of vinyl esters and acid end groups [41,42,43]. Esters containing at least one *β*-hydrogen degrade primarily through a six-membered transition state around ester linkages to give a pair of a carboxylic acid and an olefinic fragment (path 1) [42,43,44,45]. Succinic anhydride (path 2) could simply be formulated via a cyclization decomposition mechanism from succinic acid end molecules, pre-existent in the macromolecular chains or produced throughout *β*-hydrogen bond scission [41,42]. Furthermore, carboxyl end groups of larger fragments could degrade through decarboxylation during pyrolysis to produce alkyl-ended segments, such as hex-5-en-1-yl propionate (path 4). In a limited manner, α-hydrogen bond scission takes place also producing aldehydes (path 8). According to our knowledge, the mechanism of PHSu thermal degradation is illustrated for the first time.

**Scheme 2:**
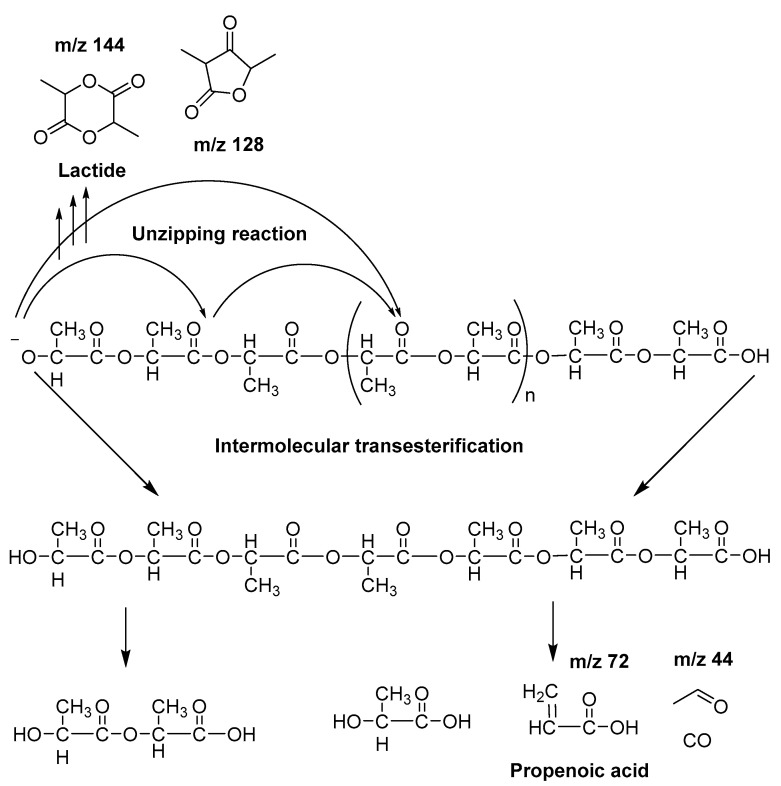
Simplified mechanism of PLA thermal degradation.

On the other hand, concerning PLA thermal degradation pathway, it is really less complex compared with PHSu. An oversimplified pathway of its thermal degradation is depicted in Scheme 2. In fact, at temperatures higher than 200 °C, intramolecular trans-esterification processes domains with the formation of lactide and cyclic oligomers, whereas cis-elimination leads to acrylic acid and oligomers. Furthermore, fragmentation provokes the final formation of acetaldehyde and CO_2_ [46]. The main pyrolytic products of PLA thermal degradation are illustrated in Table 2.

In detail, according to the chromatogram of PLA (Figure 4f), the larger intensity peak corresponds to acetaldehyde, while 2-propenoic acid (acrylic acid) and 2,3-pentanedione can also be identified. Figure 4 exhibits the presence of two peaks (R_t_ = 10.25 and 11.88 min), assigned to the fragments with m/z of 28, 43, 45 and 56. The aforementioned peaks conformed to meso-lactide and d,l-lactide structures, respectively. Peaks appearing periodically at retention times larger than 20 min were attributed to larger cyclic lactide oligomers [13,47,48].

Regarding the obtained chromatograms (Figure 4b–e), a similar pattern for the PLA-b-PHSu copolymers can be observed. In all cases, CO_2_ or acetaldehyde, owing to the carboxyl end groups of copolymers, are detected. The identified pyrolysis products of PLA-b-PHSu copolymers revealed that decomposition takes place primarily through *β*-hydrogen bond scission, forming mainly allyl and diallyl compounds at larger retention times. However, as it can be seen (Table 2), there are also some aldehydes (retention time 21.90 min) as byproducts, which may be formed through α-hydrogen bond scission [14]. As detected in the chromatogram of Figure 4b, in which the pyrolysis temperature was higher, the intensity of the recorded peaks increased, indicating that the amount of decomposition products is directly dependent on the decomposition temperature [42].

The detection of two distinct degradation steps in the TGA thermogram of PLA-b-PHSu80/20 led to the pyrolysis at both peak temperatures of its DTG. The chromatograms recorded after pyrolysis at 319 °C and 410 °C, although presenting the same peaks that correspond to the same products, exhibit some differences. Firstly, the peak presented at R_t_ = 2.8 min, corresponding to acrylic acid, can only be seen at 410 °C revealing that higher temperatures favor the cis-elimination of PLA segment. At 319 °C and from approximately R_t_ = 2.5–10 min, no peak can be observed. Furthermore, the correlation between pyrolysis temperature and α-scission products could be clearly observed, since the quantities of released aldehydes (R_t_ = 21.91 min) seemed to increase for the lower temperature of 319 °C in comparison with 410 °C. In fact, allyl and diallyl compounds are dominating at 410 °C, while at 319 °C products of α-scission are the most dominant. It is also observed that the peak at R_t_ = 34.35 at 410 °C is higher than that at 319 °C (R_t_ = 19.05 min) and it is taking place earlier. The above-mentioned statement is clear proof that both mechanisms are taking place simultaneously (*α*- and *β*-hydrogen bond scission) at the studied decomposition temperatures but to a different extent. Moreover, it is to be underlined that as the content of PHSu segment in the studied copolyesters increase, the relative peak areas of the cyclic lactides for R_t_ 10.2–11.9 min enlarge, too. In addition, the morphology of the peaks attributed to the lactide stereoisomers changed, while the two peaks exhibited a tendency to be merged into one for the sample PLA-b-PHSu80/20, especially at the pyrolysis temperature of 319 °C. As noted below, PLA degrades via several mechanisms including random chain scission inside the polymer and backbiting of the terminal hydroxyl ends [47]. The carboxyl ends promote PLA degradation by polarizing ester bonds and accelerating hydrolysis. Thus, as PHSu content increases, more carboxyl end groups are formed during pyrolysis which may catalyze the breaking of alkoxy and acyloxy bonds of the PLA counterpart and provoke the *trans*-esterification processes and the final formation of cyclic lactides [48]. Thus, it can be said that the existence of PHSu promotes the unzipping decomposition of PLA and the formation of cyclic lactides. Furthermore, at low temperatures, the thermal degradation of PLA is mainly the backbiting reaction from hydroxyl chain ends, while at higher temperatures, the thermal degradation is mainly caused by ester bonds, leading to random scission of the backbone. This fact can also boost the statement that for the sample PLA-b-PHSu80/20, which was pyrolyzed at two different temperatures, the peak intensity of lactide diastereomers was higher for the one pyrolyzed at the lowest temperature [49]. Finally, as mentioned above concerning the data of thermogravimetric analysis (Figure 2), with the increase of PHSu content the first degradation step of the studied copolyesters increases as well, supporting also the statement that this stage is mainly attributed to the formation of lactides.

### 3.4. Kinetic Analysis Based on Thermogravimetric Data—Isoconversional Methods

Thermal degradation kinetics is a handful tool to understand deeply the degradation mechanisms and analyze the thermal degradation process, which can be achieved through thermogravimetric analysis (TGA). Kinetic analysis can help scientists to calculate accurately the activation energy (E_a_) as a function of the degree of conversion (a), the pre-exponential factor (A) and the reaction mechanism (f(a)). The reaction mechanism of the polymers’ degradation is very complex, while it includes initiation, propagation and termination reactions [15]. Kinetic analysis is most commonly performed in two steps: isoconversional and model fitting. Using the isoconversional methods, the activation energy (E_a_) and the pre-exponential factor (A) are calculated, while by the model fitting method, the reaction mechanism is identified and the activation energy (E_a_) and the pre-exponential factor (A) are given. Therefore, applying these two steps in comparison, accurate results can be obtained [50]. The most popular method for kinetics based on thermal analysis is the differential isoconversional method by Friedman.

The basis of the isoconversional methods is the assumption that the conversion function f(a) does not alter with the change of the heating rate for the entire range of the degree of conversion a, thus they are called “model free” methods. Most of the kinetic methods used in kinetic analysis consider the rate of the degree of conversion a to be a function of two variables.

(1) $$\frac{\mathrm{da}}{\mathrm{dt}}=k\left(T\right)f\left(a\right),$$

where t is the time, T is the temperature, k(T) is the rate coefficient which originated from the Arrhenius law $k\left(T\right)={\mathrm{Ae}}^{-E/\mathrm{RT}}$, a is the extend of conversion and f(a) is the mathematical function denoting the reaction mechanism.

As for the differential isoconversional methods, Friedman’s method is the most common and can be obtained by applying the isoconversional principle to Equation (1). Thus, Friedman’s equation takes the form:

(2) $$\ln\left[\beta_{i}{\left(\frac{\mathrm{da}}{\mathrm{dt}}\right)}_{a,i}\right]=\ln\left[f\left(a\right)A_{a}\right]-\frac{E_{a}}{{\mathrm{RT}}_{a,i}},$$

where *A* is the pre-exponential factor and *β* is the heating rate. In order to obtain the activation energy E_a_ values, when the conversion function is constant, the slope of the straight lines of the plot ln[β_i_(da/dt)_a,i_] vs. 1/T_a,i_ should be calculated. Basically, it involves temperature measurements, which correspond to fixed a values, at different heating rates *β*. If the calculated activation energy remains approximately the same for the whole range of the degree of conversion a, a single-step reaction can be concluded with certainty.

**Figure 5:**
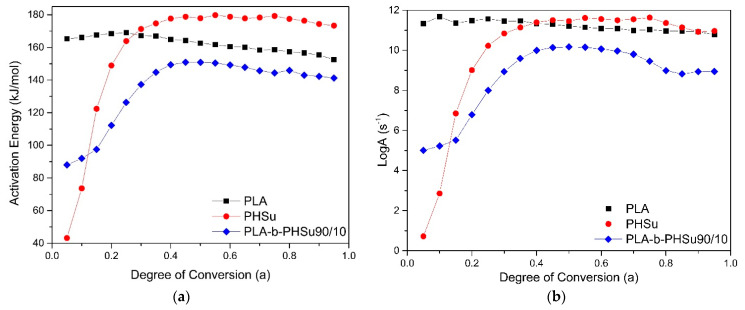
(**a**) Dependence of the activation energy (*E_a_*) and (**b**) Log(A) with the degree of conversion as calculated using Friedman’s method for PLA and PHSu and PLA90-b-PHSu90/10 copolymer.

The kinetic study and the identification of the mechanism or mechanisms of a transformation can lead to usable results only if the experimental data are reliable. Taking into consideration the TGA results, kinetic analysis was performed only on the neat PLA, PHSu and to the PLA-b-PHSu90/10 samples, in order to evaluate the mass–temperature relation, through the reactions and the mechanisms taking place during the decomposition process. We did not proceed to kinetic analysis for PLA-b-PHSu95/05 due to almost the same degradation profile with PLA. PLA-b-PHSu80/20 presents a more complicated degradation profile especially since the second degradation step is almost linear which cannot provide accurate results in the fitting process. Moreover, from Figure 2b it can be seen that the steps are in fact overlapped. Degradation kinetic analysis of PHSu and of the new PLA-b-PHSu copolymers are studied for the first time in literature. Differential methods are considered rather accurate due to the absence of mathematical approximations, compared to integral methods. Friedman isoconversional method was applied to PHSu, to PLA and to the copolymer of the PLA and PHSu with 90 wt.% and 10 wt.% proportionally. The results are presented in Figure 5.

PLA has an almost constant activation energy, around 160 kJ/mol and indicates a single-step reaction mechanism which is examined later. The almost constant E_a_ of PLA is also observed by Yuzay [17] and Das [18], who attribute PLA’s low activation energy to the low energy of non-radical backbiting ester interchange reactions involving OH chain ends. As far as PHSu is concerned, it presents a rapid increase in activation energy until 180 kJ/mol at a lower degree of conversion (a) and for a > 0.3 E_a_ remains also almost constant. The initial rapid increase refers to the low rate of mass loss during the degradation process until approximately 380 °C (Figure 1). PLA-b-PHSu90/10 affected by the presents of PHSu shows also a rapid increase in activation energy as PHSu, with the values fluctuating between 90 and 150 kJ/mol. The shape of the curve at a < 0.4 derives from the first mass loss step. The curves’ behavior of PHSu and PLA-b-PHSu90/10 indicate that the decomposition of the samples may be described by at least two-step mechanism, while that of PLA by single-step mechanism. Figure 5b presents the dependence of Log(A) with the degree of conversion (a). The three curves have a similar form with the activation energy curves.

In order to determine the nature of the mechanism or mechanisms through the comparison of the theoretical and experimental data and to confirm the number of reactions that emerged from kinetic analysis, the “model fitting method” is used. The model fitting methods are based on different models, which can define the decomposition process; including fitting different models to the degree of conversion (a) versus temperature (°C) curves and simultaneously determining the activation energy E_a_ and the pre-exponential factor A. By applying and comparing both the isoconversional methods and the model-fitting methods, the experimental data can be precisely fitted and provide accurate information about the thermal behavior of the sample. The most applied reaction models on the polymers’ thermal degradation in literature are those of the nth-order model, with only one parameter and the first-order model [15].

Therefore, all samples were heated under the rates of 5, 10, 15 and 20 °C min^−1^ and the obtained experimental data were fitted with 16 different kinetic models (Table 3) and their combinations. Initially, the model-fitting procedure includes the assumption that the samples’ decomposition can be described by a single-step mechanism, which corresponds to the main mass loss. Otherwise, if the fitting results do not fulfill the accepted requirements, it is required to proceed to experimental data fitting with a combination of two or more mechanisms.

Kinetics Neo software is a handful tool that can evaluate the experimental data by identifying them with 16 equations, either as a single mechanism or as a combination of different mechanisms. As a result, the number of different equations and parameters controlled by the program is very large and there can be many combinations with equally satisfactory results. Thus, the correlation coefficient R^2^ should be as high as 0.999.

**Figure 6:**
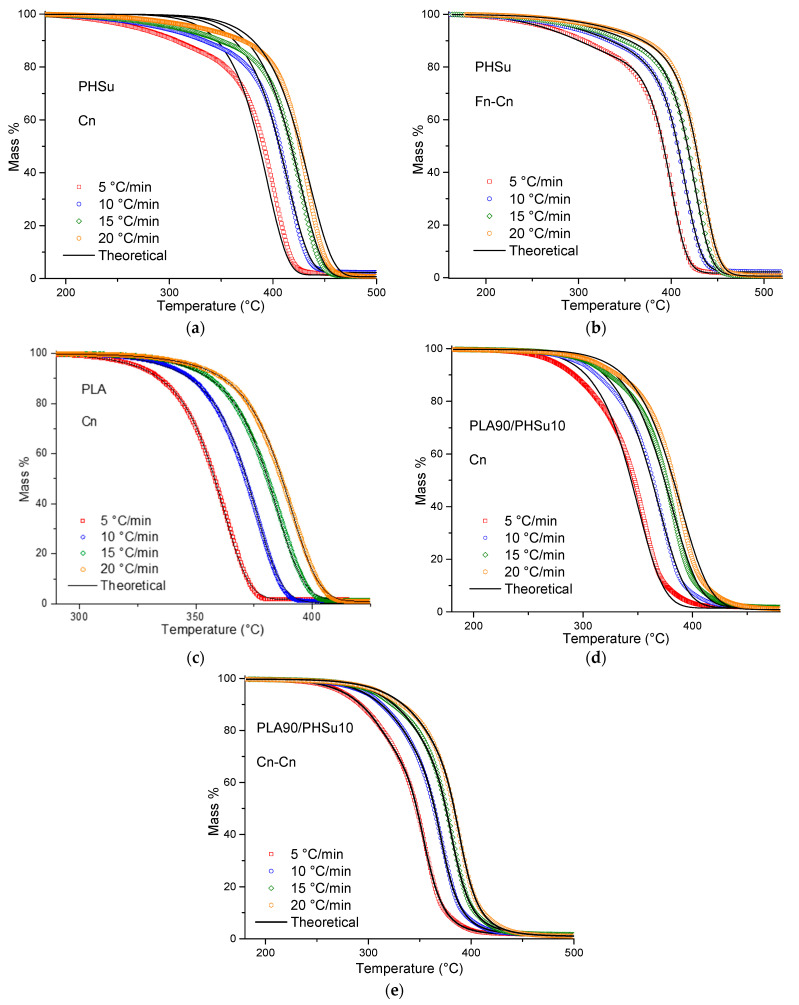
Thermal degradation of (**a**,**b**) PHSu, (**c**) PLA and (**d**—**e**) PLA-b-PHSu90/10 at different heating rates (red) 5 °C/min, (blue) 10 °C/min, (green) 15 °C/min, (orange) 20 °C/min. The colored symbols represent the experimental data, while the continuous black lines represent the fittings with different models.

The single step procedure was initially applied to the 3 samples. Only PLA (Figure 6c) was successfully fitted by a single model, which was the nth order model with autocatalysis Cn: $f\left(a\right)={\left(1-a\right)}^{n}\left(1+K_{\mathrm{cat}}\right)X$, where *X* is the extent of conversion of the autocatalytic reactions and K_cat_ is the autocatalysis rate constant. Fitting PHSu with a single step mechanism (Figure 6a) was rather inaccurate due to the low rate of mass loss at the begging of the decomposition process (R^2^ = 0.9968). Moreover, the fitting process with a single step model in PLA-b-PHSu90/10 was also not satisfied (R^2^ = 0.9993), due to the two degradation steps. Thus, and as expected from the isoconversional analysis, PHSu and PLA90/PHSu10 indicate that a two-step mechanism would be more suitable and, as a consequence, different combinations of mathematical models (consecutive or parallel) were tested. The combinations that provided the most accurate fitting results to describe the decomposition were defined by two mechanisms; Fn-Cn for PHSu and Cn-Cn for PLA-b-PHSu90/10, where Fn is an nth order: $f\left(a\right)={\left(1-a\right)}^{n}$ (Figure 6b,e). In PLA-b-PHSu90/10, both mechanisms are autocatalysis; the first that takes place at low temperatures corresponds to small mass loss and the second with higher activation energy to the main degradation step.

The values of *E_a_* and logA that resulted from the “model fitting method” (Table 4) are in the same range and maintain the same trend as those obtained from the isoconversional method (Figure 5).

As it was observed, two equations for PLA-b-PHSu90/10 were used to fit the degradation plot and thus we believe that in order to fit the degradation curve of PLA-b-PHSu80/20 at least three equations in proportion would need to be used. The number of different combinations of equations (reaction mechanisms) and at least 24 different parameters would provide a significant number of different combinations of equations and parameters with equally good identification results. Unfortunately, in such complicated samples, it is extremely inaccurate to choose the right combination that will have a physical meaning. In most cases, literature does not include such complicated and inaccurate identifications and as a consequence a full study of those mechanisms.

## 4. Conclusions

PLA-b-PHSu copolymers have been successfully synthesized and, from mechanical tests, it was found that all copolymers have a slightly lower tensile strength at break but a significant increase at elongation at break was observed in the PLA-b-PHSu80/20. Thermogravimetric analysis (TGA) in conjunction with pyrolysis-gas chromatography/mass spectrometry (Py-GC/MS) was successfully applied to study the decomposition kinetic and mechanism of PLA-b-PHSu copolymers. PLA-b-PHSu80/20 showed the most differences. Both isoconversional methods and “model fitting methods” in comparison revealed rather accurate results (E,logA). PLA is the only one that is described with a single step reaction (Cn), while PHSu and PLA-b-PHSu90/10 with a two-step reaction (Fn-Cn the PHSu and Cn-Cn the PLA-b-PHSu90/10). From PY-GC/MS, it was found that the principal thermal degradation mechanism of PHSu is the *β*-hydrogen bond scission forming vinyl and carboxyl end groups, while cyclization from succinic acid end molecules takes place also producing anhydrides. Aldehydes can be also produced by *α*-hydrogen bond scission. PLA thermal degradation was also studied, and it was found that at temperatures higher than 200 °C intramolecular *trans*-esterification processes domains with the formation of lactide and cyclic oligomers, whereas *cis*-elimination leads to acrylic acid and oligomers. The addition of PHSu in copolymers seems to promote the formation of cyclic lactides, which takes place in progressively lower temperatures than neat PLA and this was proved from Py-GC/MS analysis of PLA-b-PHSu80/20 copolymer.

## Figures and Tables

**Table 1:** Temperatures of the maximum degradation rate.

Sample	T_d,max_ (°C)
PHSu	432.3
PLA	390.5
PLA-b-PHSu95/05	331.9, 393.7
PLA-b-PHSu90/10	324.8, 387.9
PLA-b-PHSu80/20	319.4, 410.9

**Table 2:** Possible pyrolysis products evolved during thermal decomposition of PLA, PHSu and their copolymers, at different temperatures as identified with mass spectrometry based on the Py-GC chromatograms.

Peak No.	PLA	PLA-b-PHSu 95/05	PLA-b-PHSu 90/10	PLA-b-PHSu 80/20		PHSu	Mw (amu)	Possible Product
	Pyrolysis Temperature							
	390 °C	394 °C	388 °C	319 °C	410 °C	432 °C		
			R_t_ (min)					
1	-	-	1.19	-	1.19	1.18	44	CO_2_
2	1.27	1.32	-	-	1.36	-	56	acetaldehyde 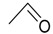
3	1.90	-	1.88	-	1.82	-	100	2,3-pentanedione 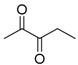
4	2.85	2.86	2.90	-	2.74	-	72	acrylic acid 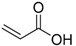
5	-	-	-	-	3.44	3.38	82	1,5-hexadiene 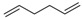
6	-	-	7.82	-	7.80	-	100	succinic anhydride 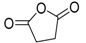
7	-	8.12	8.15	-	8.12	8.17	117	1,6-hexanediol 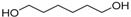
8	-	-	-	-	8.40	8.43	118	succinic acid 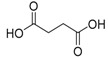
9	10.25 11.88	10.18 11.63	10.44 12.31	10.33 12.06	10.27 12.10	-	144	meso-Lactide (3,6-dimethyl-1,4-dioxane-2,5-dione) 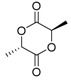 d,l-Lactide 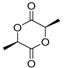
10	-	-	-	-	16.39	16.41	146	4-hydroxybutyl propionate 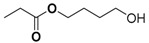
11	-	20.02	20.07	20.06	20.16	20.14	186	hex-5-en-1-yl 4-hydroxybutanoate 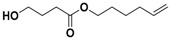
12	21.91	21.91	21.92	-	21.93	-	202	PLA trimer *or* 7-oxoheptyl 2-hydroxypropanoate 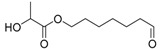
13	-	-	28.35	28.347	28.38	28.483	218	4-((6-hydroxyhexyl)oxy)-4-oxobutanoic acid 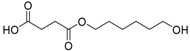
14	-	-	-	34.35	34.33	34.60	272	4-((6-(acryloyloxy)hexyl)oxy)-4-oxobutanoic acid 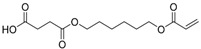
15	-	-	-	-	40.61	40.72	302	4-oxo-4-((6-((4-oxobutanoyl)oxy)hexyl)oxy)butanoic acid 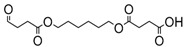
16	-	-	45.05	45.06	45.08	45.08	318	bis(6-hydroxyhexyl) succinate 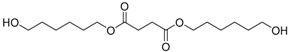
17	-	46.76	46.83	46.84	46.87	46.82	400	4-((6-((4-(hex-5-en-1-yloxy)-4-oxobutanoyl)oxy)hexyl)oxy)-4-oxobutanoic acid 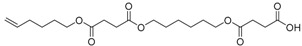

**Table 3:** Reaction models and their mathematical explanation.

Kinetic Model	Symbol	f(a)
**n-order reactions**		
First order	F1	1−a
Second order	F2	${\left(1-a\right)}^{2}$
n^th^ order	Fn	${\left(1-a\right)}^{n}$
**Diffusion**		
1-D diffusion	D1	1/2a
2-D diffusion	D2	${\left[-\ln(1-a\right)]}^{-1}$
3-D diffusion-Jander	D3	$3/2{\left(1-a\right)}^{2/3}[1-{\left(1-a\right)}^{1/3}]$
3-D diffusion-Ginstling–Brounshtein	D4	$3/2{\left[{\left(1-a\right)}^{-1/3}-1\right]}^{-1}$
**Phase-boundary reactions**		
Contracting area	R2	$2{\left(1-a\right)}^{1/2}$
Contracting volume	R3	$3{\left(1-a\right)}^{2/3}$
Prout–Tompkins	B1	a(1−a)
expanded Prout–Tomplins	Bn	${\left(1-a\right)}^{n}a^{m}$
nth order with autocatalysis	C1	(1−a)(1+Kcat X)
nth order with autocatalysis	Cn	${\left(1-a\right)}^{n}\left(1+\mathrm{Kcat}\;X\right)$
**Nucleation and nuclei growth**		
Avrami–Erofeev	A2	$2\left(1-a\right){\left[-\ln\left(1-a\right)\right]}^{1/2}$
Avrami–Erofeev	A3	$3\left(1-a\right){\left[-\ln\left(1-a\right)\right]}^{2/3}$
Avrami–Erofeev	An	$n\left(1-a\right){\left[-\ln\left(1-a\right)\right]}^{\left(n-1\right)/n}$

**Table 4:** Calculated values of activation energy (*Ea*), pre-exponential factor (A), reaction order (*n*), branching rate constant (*Kcat*) and correlation coefficient (R^2^) for PLA, PHSu and their copolymer.

**1st Step**						
**Sample**	**Mechanism**	**E_a_ (kJ/mol)**	**logA (s^−1^)**	**logK_cat_**	**React. Order n**	**R^2^**
PHSu	Fn	48	1.7	-	0.6	0.99994
PLA	Cn	161	10.6	0.8	1.0	0.99990
PLA-b-PHSU90/10	Cn	91	5.6	0.01	0.8	0.99997
**2nd Step**						
**Sample**	**Mechanism**	**E(kJ/mol)**	**logA (s^−^)**	**logK_cat_**	**React. Order n**	**R^2^**
PHSu	Cn	173	10.9	0.3	1.2	0.99994
PLA-b-PHSU90/10	Cn	146	9.4	0.9	1.8	0.99997

## Data Availability

The data presented in this study are available on request from the corresponding author.
